# Can the Mucosa Heal Histologically in Celiac Disease? What are the Indicators of Remission in Celiac Patients?

**DOI:** 10.5152/tjg.2025.24518

**Published:** 2025-04-07

**Authors:** Berat Ebik, Ferhat Bacaksiz, Mustafa Zanyar Akkuzu, Ahmet Yavuz, Ümit Karabulut, Muhsin Kaya

**Affiliations:** 1 Division of Gastroenterology, University of Health Sciences, Diyarbakır Gazi Yaşargil Education and Research Hospital, Diyarbakır, Türkiye; 2 Department of Gastroenterology, Dicle University School of Medicine, Diyarbakır, Türkiye

**Keywords:** Celiac disease, remission status, villous atrophy

## Abstract

**Background/Aims:**

The effect of a gluten-free diet on the small intestinal mucosa in patients with celiac disease (CD) is unclear. The occurrence of mucosal healing and the permanence of villous atrophy in celiac patients under a gluten-free diet were investigated.

**Materials and Methods:**

The symptoms, laboratory values, autoantibody levels, and endoscopic mucosal biopsy samples of 115 celiac patients, 56 of whom were in remission at different time periods since diagnosis, were examined.

**Results:**

Although 61.8% of patients said they were compliant with the gluten-free diet, 48.7% were in remission. Sixty-seven percent (n = 77) of patients with CD reported at least one symptom. Only weight loss was associated with disease activity (*P* = .019). While only 5 (8.9%) of the patients in remission were endoscopically and histopathologically normal, villous atrophy was detected in 69.9% of the patients. Villous atrophy was present in 86% of patients within the first 5 years of diagnosis, while this rate was 59% in patients older than 10 years. Logistic regression analysis showed that compliance with diet (odds ratio [OR]: 4.39; *P* < .001) and normal endoscopic appearance of the mucosa (OR: 13.01; *P* < .001) were the strongest predictive factors of remission.

**Conclusion:**

Diet compliance and remission rates of celiac patients increase over time. However, histologically, intraepithelial lymphocytosis almost never improves, and villous atrophy persists in most patients even if they comply with the diet. Although the asymptomatic nature of the patients, their autoantibody titers, and their dietary compliance are considered important indicators of remission, these parameters do not clearly reflect the histological status of the mucosa.

**Main Points**

- Celiac patients’ compliance with the diet has increased over the years.
- A gluten-free diet reduces symptoms and causes a decrease in autoantibody levels.
- In the majority of celiac patients, mucosal histological improvement does not occur even if they comply with the diet.

## Introduction

Celiac disease (CD) is a disease that is triggered by gluten intake in genetically predisposed individuals and causes a wide range of intestinal and extraintestinal symptoms and diseases, including malabsorption, growth and developmental retardation, and vitamin and mineral deficiencies, as a result of small intestinal mucosal damage. The only currently known treatment for CD is a lifelong gluten-free diet. A gluten-free diet is thought to reduce duodenal mucosal inflammation, the main trigger of symptoms.^1^ ^2^

A gluten-free diet prevents complications such as anemia, diarrhea, abdominal pain, and vitamin deficiencies while reducing tissue transglutaminase antibody levels. However, literature data indicating that a gluten-free diet improves mucosal damage, especially villus atrophy, is contradictory. Although it is classically stated that mucosal healing occurs within 6-12 months after starting a gluten-free diet,^3^ many studies on this subject indicate that villous atrophy and mucosal healing do not occur in all patients, and the exact timing of this cannot be predicted in many patients.^4^ ^5^

This study has 2 main objectives. The first is determining how many patients who follow the diet and are in remission experience mucosal healing and when. Also, the factors that affect remission in celiac patients have been investigated. In addition, how the symptoms frequently expressed by patients in the outpatient clinic were related to their remission status was examined.

## Materials and Methods

This study is a prospective cohort study. A total of 115 patients diagnosed with CD who were admitted to the gastroenterology outpatient clinic of Gazi Yaşargil Training and Research Hospital between January 2023 and March 2024 were included in the study.

### Study Protocol and Ethical Situation

Patients were informed about the study before being accepted into the study. Verbal and written informed consent was obtained from patients who agreed to participate in the study. After explaining the purpose of the study, in addition to routine blood tests, patients underwent endoscopy and duodenal biopsy for histopathological evaluation. This study was approved by the Clinical Research Ethics Committee of University of Health Sciences, Gazi Yasargil Education and Research Hospital, with decision number 264 dated December 9, 2022.

### Inclusion Criteria for the Study

Patients were accepted into the study if they had been diagnosed with CD for at least 1 year (those who had previously tested positive for celiac autoantibodies (anti-transglutaminase IgA or anti-endomysium IgA) and were diagnosed with CD by endoscopic biopsy^6^) and were older than 16 years of age and declared that they accepted the study protocol.

### Exclusion Criteria for the Study

Patients with newly diagnosed CD, patients with another chronic disease along with CD, patients who had undergone stomach, duodenum, and jejunum surgery or resection, patients who neglected their periodic follow-ups and had incomplete laboratory tests, and patients who did not fill out the symptom and complaint form were not included in the study.

### Upper Gastrointestinal Endoscopy and Duodenal Biopsy

Among celiac patients, upper gastrointestinal endoscopy was performed on patients who were determined to be in remission based on clinical and laboratory data. At least 4 biopsy samples were taken from the distal part of the second duodenum, and 2 biopsy samples were taken from the bulb. Samples were fixed in formalin solution for 24 hours, paraffin blocks were prepared after routine pathological procedures, and 5 μm sections were prepared with a standard microtome. Preparations were stained with Giemsa and Hematoxylin-Eosin as standard and examined under a light microscope at 200 magnification by pathology specialists in the hospital. Duodenal mucosa biopsy samples were evaluated according to the modified Marsh–Oberhuber classification.^7^

### Anti-Tissue Transglutaminase Antibodies

Venous blood samples were taken from all patients after at least 8 hours of fasting, and antissue transglutaminase antibody (tTG) tTG IgA and tTG IgG antibodies were studied by the micro-ELISA method (IMMCO 42 diagnostics ImmuLisaTM Buffalo, NY, USA). Strips coated with human tissue transglutaminase enzyme were compared with plasma samples to be tested. The resulting color change was converted into numerical results by photometric analysis. In photometric analysis, results >10 U/mL were considered positive, and results below 10 U/mL were considered negative.^8^

### Evaluation of Symptoms

In addition to laboratory data, the current symptoms of all patients included in the study were also questioned. Complaints such as abdominal pain, diarrhea, gas-bloating, and weight loss were recorded and statistically examined to determine whether these complaints were an indication of not being in remission.

### Other Laboratory Tests

When celiac patients came for a check-up, hemogram, ferritin, iron, iron-binding capacity, folic acid, vitamin B12, vitamin D, calcium, phosphorus, magnesium, albumin, glucose, urea, creatinine, sodium, potassium, liver function tests, parathyroid hormone, and Dual Energy X-RAY Absorptiometry (DEXA) to measure bone mineralization, thyroid hormones, and celiac autoantibody levels were checked as recommended by the guidelines.^9^

The body mass index (BMI) of the patients was calculated by dividing the body weight by the square of the height in meters (BMI = kg/m^2^).^10^

### Evaluation of Remission

Patients who were compliant with gluten-free diet treatment were asymptomatic and had antitransglutaminase levels below the cut-off value of 10 U/mL were considered to be in remission and had asymptomatic CD. Patients who were not compliant with the diet had symptoms of malabsorption, and laboratory parameters of anemia, vitamin and mineral deficiencies, and antitransglutaminase levels >10 U/mL were considered to have symptomatic CD.^11^

Celiac patients were divided into 2 groups: those in remission and those not. Demographic and laboratory data of both groups of celiac patients were compared. It was statistically examined which symptoms each group had and whether patients who were not in remission had more symptoms.

The main purpose of this study was the histopathological examination of the duodenal mucosa. The proportion of patients in remission on a gluten-free diet who continued to have mucosal damage and villous atrophy in the small intestine and its relationship with time was examined.

Patients were divided into 3 groups according to the period in which they were diagnosed. These groups were: patients within the first 5 years of diagnosis, patients between 5 and 10 years, and patients with a diagnosis older than 10 years. Factors such as dietary compliance, remission status, and autoantibody positivity were examined among these 3 groups. The aim was to reveal data on whether remission in celiac patients increased over the years and whether the patients’ skills in recognizing their disease and learning to live with it improved. Finally, the factors that are effective in maintaining remission in celiac patients were statistically examined.

### Statistical Analysis

Kolmogorov–Smirnov, Shapiro–Wilk test, coefficient of variation, skewness and kurtosis methods were used to check the normal distribution of patient data. While mean and standard deviation values were stated for continuous variables, categorical variables were expressed as percentages. Independent *t*-test or Mann–Whitney *U* test was used to determine the difference in age, body mass index, dietary compliance, and laboratory parameters between patients with CD in remission and those not in remission. The Pearson chi-square test was used to determine the differences in gender and the presence of symptoms between the 2 groups. To measure the effect of known disease duration on diet, remission status, and autoantibody positivity, the 1-way ANOVA test was applied to parameters with homogeneous variances, and Welch ANOVA and Kruskal–Wallis tests were applied to parameters with non-normal distribution and inhomogeneous variances. Hierarchical logistic regression analysis was performed to evaluate the factors affecting remission in celiac patients. Correlation coefficients and odds ratios were calculated along with 95% CI values. All tests were two-sided, and *P*-value <.05 was considered statistically significant. Statistical analyses were performed using SPSS 24.0 for Windows (IBM SPSS Corp.; Armonk, NY, USA) package program.

## Results

Of the 115 celiac patients included in the study, 72.2% (n = 83) were female and 27.8% (n = 32) were male. While 48.7% (n = 56) of the patients were in remission, 51.3% (n = 59) were not in remission. No difference was found in gender distribution between CD in remission and non-remission (*P* = .406). The mean age of the patients was 29.1 years. The mean age of CD patients in and out of remission was similar (*P* = .475). The mean time for diagnosis of CD was 10.5 years in patients in remission and 7.6 years in patients not in remission (*P* = .002). The body mass index of celiac patients in remission was higher than those not in remission (21.1 kg/m^2^ ± 3.2 vs. 19.6 kg/m^2^ ± 2.9; *P* = .015). 61.8% of celiac patients stated that they were on a gluten-free diet. This rate was 80.4% in patients in remission and 44.1% in patients not in remission (p<0.001). The mean hemoglobin value in celiac patients in remission was 14.0 g/dL, while it was 12.8 g/dL in patients not in remission (*P* = .002). Similarly, the mean ferritin level was lower in patients with CD who were not in remission (72.2 µg/L vs. 44.4 µg/L; *P* < .001). Although vitamin B12 levels were similar in both groups (*P* = .322), folic acid levels were higher in patients in remission (*P* < .001). The mean vitamin D level in celiac patients in remission was 18.6 ng/mL, while it was 13.3 ng/mL in patients not in remission (*P* = .006). Although symptomatic hypocalcemia was not evident (only 1 case), the mean calcium level was lower in non-remission celiac patients (*P* < .001). The mean parathyroid hormone level was higher in patients with CD not in remission (76.3 pg/mL vs. 54.4 pg/mL; *P* = .001). The mean magnesium level in both groups was similar (Table 1).

While 33% (n = 38) of celiac patients did not report any symptoms, 67% (n = 77) reported at least one symptom. The most frequently reported symptoms were abdominal pain (35.6%) and gas-bloating (36.5%). Abdominal pain was present in 25% of patients in remission, while it was present in 37.3% of patients not in remission (*P* = .157). On the other hand, although gas and bloating complaints were reported more in patients not in remission (42.4% vs. 30.4%), it was not significant (*P* = .126). Diarrhea was present in 14.8% (n = 17) of the celiac patients, but no difference was found between patients in remission and those not in remission (*P* = .175). The proportion of patients with celiac disease who stated that they had experienced weight loss was 29.6%. While the rate of patients reporting weight loss in celiac disease in remission was 19.6%, this rate increased to 39.0% in patients not in remission (p=0.019). (Table 2).

**Figure 1.**
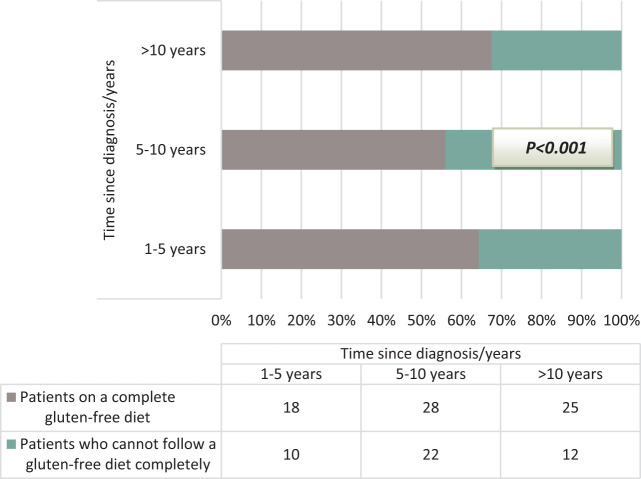
Known duration of disease and dietary compliance rate in Celiac patients.

While 64.2% (n = 18) of the patients within the first 5 years of the diagnosis of the disease complied with the diet, this rate was 56% (n = 28) in those within 5-10 years and 67.5% (n = 25) in patients older than 10 years (Figure 1).

**Figure 2.**
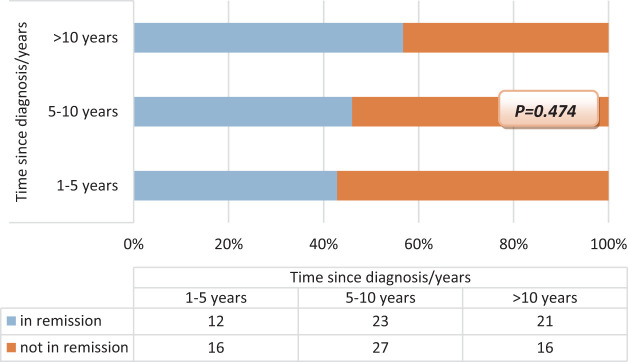
Known duration of disease and remission rates in Celiac patients.

The relationship between the known time since the diagnosis of the disease and the rate of remaining in remission is examined, and although this rate increased over the years, it did not gain statistical significance due to the number of patients. While 42.8% of those with a known disease duration of less than 5 years were in remission, this rate was 46.0% in those with 5-10 years, and 56.7% of celiac patients with more than 10 years were in remission (*P* = .474) (Figure 2).

**Figure 3.**
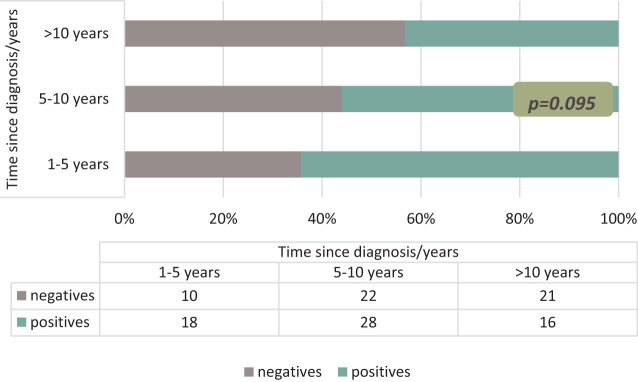
The relationship between the time elapsed since diagnosis and the rate of positive detection of anti-transglutaminase antibodies in Celiac patients.

Similarly, anti-transglutaminase antibody positivity, one of the indicators of remission status, decreased as the known duration of the disease increased. While anti-tTg IgA antibody was positive in 64.2% of those with the disease in the first 5 years, this rate was 56.0% in those with the disease between 5 and 10 years and 43.2% in patients with the disease for more than 10 years (*P* = .095) (Figure 3).

**Figure 4.**
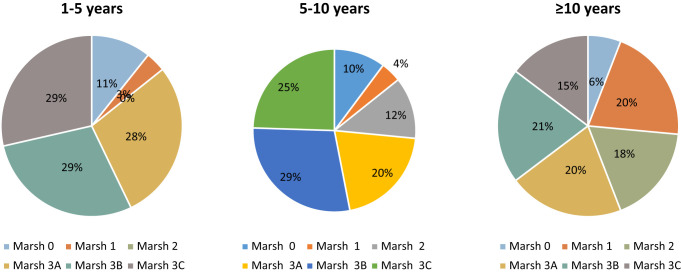
Distribution rates of histopathological findings with known duration of disease in celiac patients in remission.

Endoscopic findings were found to be normal in only 5 (8.9%) of 56 celiac patients in remission who underwent endoscopy and had the opportunity for histopathological examination. Although no change was detected in the number of intraepithelial lymphocytes in celiac patients over the years, villus atrophy was found to be less common. Villous atrophy was present in 86% of celiac patients within the first 5 years of diagnosis, while villous atrophy was present in 72% of those within 5-10 years. This rate was 59% in celiac patients older than 10 years. Villous atrophy was detected in 69.9% of the celiac patients in remission (*P* = .054) (Figure 4).

Logistic regression analysis showed that the presence of symptoms, diet compliance, and normal endoscopic appearance were effective factors on remission. Age, gender, and known duration of disease were found to have no effect on remission. The rate of patients who did not have any symptoms such as abdominal pain, diarrhea, weight loss, or gas-bloating was 3 times higher than those who had at least one symptom (*P* = .007). The rate of remission in patients who stated that they followed the diet was 4.3 times higher than those who did not follow the diet (*P* < .001). Patients with normal endoscopic appearance were 13 times more likely to remain in remission than patients with abnormal endoscopic appearance (*P* < .001). Multivariate analysis showed that full compliance with the diet (odds ratio [OR]: 3.19; *P* < .001) and endoscopic appearance of the mucosa (OR: 8.92; *P* < .001) were strong predictors of remission (Table 3).

## Discussion

Although the majority of the patients stated that they were paying attention to their diet, the number of celiac patients in remission was less than 50%. It was observed that complaints such as abdominal pain, diarrhea, gas, and bloating, which they frequently mentioned during outpatient clinic check-ups, were not related to remission. Only weight loss stood out as a finding indicating disease activity. The fact that there was no difference in these symptoms in both groups showed that other factors, especially irritable bowel syndrome (IBS) and dyspepsia, also had an effect. The symptoms of these patients overlapped more with functional dyspepsia and IBS. Many of these symptoms, which are not specific to CD, are also known to be symptoms of IBS. Studies indicate that CD may overlap with IBS. In a meta-analysis including approximately 4000 celiac patients, the frequency of IBS was found to be higher in celiac patients than in control groups.^12^

Over the years, it has been observed that the increase in patients’ knowledge and experience regarding their diseases is reflected in their behavior. As the known duration of the disease increases, the number of patients compliant with the diet also increases. This was reflected in an increase in the number of patients in remission and an increase in the number of patients with negative tissue transglutaminase antibody levels. Although the tissue transglutaminase antibody level is an indicator of compliance with the diet, it is not an indicator of mucosal healing, especially villous atrophy.^13^ ^14^

It can be said that the past years have been especially good for celiac patients who follow the diet. Because the older the known duration of the disease, the lower the rate of villous atrophy. A study conducted in this context stated that the more severe the villous atrophy at the time of diagnosis, the longer the mucosal healing period.^15^ It is possible to say that some of the celiac patients who have more mucosal damage and villous atrophy in the early stages have improved these findings over time. However, it cannot be said that this is true for all patients. In nearly half of patients, villous atrophy probably persists for life. Approximately 70% of the patients who were followed in remission had villous atrophy. In a study in which 465 celiac patients were followed on a long-term gluten-free diet, it was observed that villous atrophy regressed in only 8% of the patients in the long term.^16^ In another study conducted with 381 celiac patients, it was stated that histological healing was difficult even under a gluten-free diet.^17^ In their study, Wahab et al^18^ reported that only one-third of celiac patients achieved complete mucosal healing and that this could take a very long time. In a study conducted in Spain, 53% of the patients were found to have negative tissue transglutaminase antibodies in a 2-year follow-up, and in a similar study conducted in the United States, 90% of 93 celiac patients who were followed for 6 years on a gluten-free diet had negative mucosa, but only 1% of the patients had histologically normal mucosa.^19^ ^20^ This shows that enteropathy in CD is permanent.

In a study claiming the opposite, it was stated that 81% of 105 children with CD had histological improvement after a 1-year gluten-free diet. Patients who do not recover histologically have been associated with IgA deficiency.^21^ Studies indicate that mucosal healing is faster in children.

In addition to villous atrophy, intra-epithelial lymphocytosis was detected in almost all of the patients. Studies in this direction show that intraepithelial lymphocytosis in celiac patients is a permanent mucosal abnormality independent of infectious agents such as *Helicobacter pylori*.^22^ This situation shows us that even if a patient is asymptomatic, has normal laboratory values, is celiac autoantibody negative, and compliant with the diet, complete recovery in a celiac patient can only be demonstrated through duodenal mucosa biopsies.

In summary, although the patients’ asymptomatic status, low autoantibody titers, and compliance with a gluten-free diet are considered important indicators for remission, these parameters do not clearly reflect the histological status of the mucosa and are not more valuable than endoscopic biopsy in evaluating remission. This research revealed that the most valuable indicator of remission in celiac patients is intestinal histology. However, at this time it can be said that mucosal healing with a gluten-free diet is not a rational target for remission. This could only be a target for molecules to be developed in the future.

## Figures and Tables

**Table 1.** Demographic and Laboratory Data of Celiac Patients Included in the Study

Parameters	Study Population (n = 115)	In Remission (n = 56)	Not in Remission (n = 59)	*P*
Age	29.1 ± 10.6	29.9 ± 11.8	28.4 ± 9.4	.475
Gender				
F	83 (72.2%)	38 (67.9%)	45 (76.3%)	.406
M	32 (27.8%)	18 (32.1%)	14 (23.7%)	
BMI (kg/m^2^)	20.3 ± 3.1	21.1 ± 3.2	19.6 ± 2.9	**.015**
Time since diagnosis/years	9.1 ± 5.0 (1-30)	10.5 ± 5.7	7.6 ± 3.8	**.002**
Diet compliance Diet compatible Incompatible with diet	71 (61.8%) 44 (38.2%)	45 (80.4%) 11 (19.6%)	26 (44.1%) 33 (55.9%)	**<.001**
HGB (g/dL)	13.4 ± 1.9	14.0 ± 1.6	12.8 ± 2.0	** .002**
Ferritin (µg/L)	30.0 (2.5-217)	72.2 (3.2-217)	44.4 (2.5-87)	**<.001**
Folate (ng/mL)	6.8 ± 4.1	8.6 ± 4.5	5.1 ± 3.0	**<.001**
Vitamin B12 (ng/L)	364 ± 136	377 ± 132	352 ± 139	.322
Vitamin D (ng/mL)	15.9 (2.8-88.2)	18.6 (4.7-88.2)	13.3 (2.8-83.0)	**.006**
Calcium (mg/dL)	9.2 ± 0.5	9.5 ± 0.5	9.0 ± 0.5	**<.001**
PTH (pg/mL)	65.6 (22.6-471)	54.4 (25.5-140)	76.3 (22.6-471)	**.001**
Magnesium (mg/dL)	1.8 ± 0.1	1.8 ± 0.2	1.8 ± 0.2	.922

BMI, body mass index; HGB, hemoglobin; PTH, parathyroid hormone.

**Table 2.** Remission Status of Celiac Patients Based on Their Symptoms

Symptoms, n (%)	Study Population	In Remission	Not in Remission	*P*
Abdominal pain Yes No	36 (31.3) 79 (68.7)	14 (25.0) 42 (75.0)	22 (37.3) 37 (62.7)	.157
Diarrhea Yes No	17 (14.8) 98 (85.2)	6 (10.7) 50 (89.3)	11 (18.6) 48 (81.4)	.175
Weight loss Yes No	34 (29.6) 81 (70.4)	11 (19.6) 45 (80.4)	23 (39.0) 36 (61.0)	**.019**
Gas and bloating Yes No	42 (36.5) 73 (63.5)	17 (30.4) 39 (69.6)	25 (42.4) 34 (57.6)	.126

**Table 3.** Logistic Regression Analysis of Main Factors Affecting Remission Status in Celiac Patients

Parameter	Univariate Analysis			Multivariate Analysis		
	OR	95% Cl	*P*	OR	95% CI	*P*
Age*	1.17	0.55-2.18	.677			
Gender**	1.52	0.66-2.46	.315			
Duration of illness***	0.62	0.28-1.36	.235			
Presence of symptoms****	3.01	1.33-5.78	.007	1.68	0.78-3.62	.182
Dietary compliance	4.39	1.96-8.80	<.001	3.19	2.27-7.97	<.001
Endoscopic view*****	13.01	7.62-23.68	<.001	8.92	6.41-20.1	<.001

CI, confidence interval; OR, odds ratio.

*<30 years of age reference.

**Female gender reference.

***Those with known disease for more than 10 years reference.

****Those without any symptoms such as abdominal pain, diarrhea, bloating, weight loss reference.

*****Those with normal endoscopic appearance reference.
